# An Integrated Clinical‐Radiomics‐Deep Learning Model Based on ^18^F‐FDG PET/CT for Predicting EGFR Mutation Status in Lung Adenocarcinoma

**DOI:** 10.1002/cam4.71370

**Published:** 2025-11-20

**Authors:** Yun Wang, Zhaoqing Chen, Jing Li, Yuhuang Cai, Chengyang Sun, Jingjing Zhang, Marcus Hacker, Xiang Li, Heqing Yi

**Affiliations:** ^1^ Department of Nuclear Medicine Zhejiang Cancer Hospital Hangzhou Zhejiang China; ^2^ School of Information Engineering and Internet of Things Huzhou Vocational Technical College Huzhou Zhejiang China; ^3^ Department of Nuclear Medicine Aksu Prefecture First People's Hospital Aksu Xinjiang China; ^4^ Postgraduate Training Base Alliance of Wenzhou Medical University (Zhejiang Cancer Hospital) Hangzhou Zhejiang China; ^5^ Division of Nuclear Medicine, Department of Biomedical Imaging and Image‐Guided Therapy Medical University of Vienna Wien Austria; ^6^ Department of Nuclear Medicine Beijing Chest Hospital, Capital Medical University Beijing China; ^7^ Key Laboratory of Prevention, Diagnosis and Therapy of Upper Gastrointestinal Cancer of Zhejiang Province Hangzhou Zhejiang China

**Keywords:** ^18^F‐FDG PET/CT, deep learning, epidermal growth factor receptor, lung adenocarcinoma

## Abstract

**Background:**

An integrated model combining clinical variables, radiomic features, and deep learning was developed to predict EGFR mutation status in patients with lung adenocarcinoma based on pretreatment ^18^F‐FDG PET/CT imaging.

**Methods:**

In this retrospective study, data from 218 patients—including PET/CT images, EGFR mutation status, and clinical characteristics—were analyzed. Three predictive models were constructed: a clinical model (C), a clinical‐radiomics model (CR), and a clinical‐radiomics‐deep learning model (CRD).

**Results:**

The CRD model integrated screened clinical features, as well as ConvNext‐based deep learning scores and radiomic scores selected via LASSO regression. It exhibited significantly superior predictive performance to the C model (AUC = 0.599; DeLong test: *Z* = –3.522, *p* < 0.001, corrected *p* = 0.001) and the CR model (AUC = 0.739; DeLong test: *Z* = –2.197, *p* = 0.028, corrected *p* = 0.028), with an AUC of 0.821 for the CRD model. Calibration curves and decision curve analysis confirmed its robustness and potential clinical benefit. A nomogram based on the CRD model was established, enabling individualized risk prediction of EGFR mutation.

**Conclusions:**

This study highlights the potential of integrating clinical, radiomic, and deep learning features as a noninvasive approach for accurately predicting EGFR mutation status in lung adenocarcinoma.

**Abbreviations**
5‐FCV: five‐fold cross validation
ACC: accuracy
AUC: area under the curve
C model: clinical model
CI: confidence interval
Cli: clinical feature
Con: ConvNeXt features
CR model: clinical‐radiomics model
CRD model: clinical‐radiomics‐deep learning model
Den: DenseNet169 features
DT: decision tree
Eff: EfficientNet features
EGFR: epidermal growth factor receptor
LR: logistic regression
NPV: negative predictive value
PPV: positive predictive value
Rad: radiomic features
Res: ResNet50 features
RF: random forest
ROC: receiver operating characteristic
SEN: sensitivity
SPE: specificity
SUV_max_
: maximum standardized uptake value
SVM: support vector machine
Swin: Swin Transformer features
TLG: total lesion glycolysis
XGB: XGBoost

## 1 Introduction

Lung cancer is the second most frequently diagnosed cancer worldwide, with non‐small cell lung cancer (NSCLC) comprising approximately 80%–85% of all cases [1, 2]. Among NSCLC subtypes, lung adenocarcinoma is the most prevalent. Targeted therapies against the epidermal growth factor receptor (EGFR) have markedly improved treatment outcomes for patients with EGFR‐mutant lung adenocarcinoma [3, 4]. Currently, the gold standard for determining EGFR mutation status is molecular testing of tumor tissue obtained through needle biopsy or surgical resection. However, these procedures are invasive, time‐consuming, and may yield inaccurate results due to intratumoral heterogeneity [5]. Therefore, there is a critical need for noninvasive, rapid, and reliable approaches to predict EGFR mutation status.

Genotypic heterogeneity influences the tumor microenvironment, including metabolic activity, and can be captured by medical imaging. ^18^F‐fluorodeoxyglucose (^18^F‐FDG) positron emission tomography (PET), which reflects glucose metabolism and is regulated by EGFR signaling in mutant cells, is widely used in lung cancer imaging [6]. The integrated ^18^F‐FDG PET/CT modality is routinely employed for diagnosis, staging, prognosis assessment, and treatment monitoring in clinical practice. Traditionally, studies using ^18^F‐FDG PET/CT to predict gene mutations have relied on visual interpretation or conventional semiquantitative metrics, such as standardized uptake values (SUVs), often neglecting CT‐derived information and image texture features that reflect tumor heterogeneity [7, 8, 9]. With the emergence of medical artificial intelligence, it is now feasible to rapidly and noninvasively identify surrogate biomarkers for EGFR genotyping. Radiomics, which quantitatively analyzes tumor phenotypes through high‐throughput feature extraction from medical images, offers a promising strategy for such applications.

Previous studies have demonstrated that both semantic CT features and quantitative radiomic metrics can aid in predicting EGFR mutation status [10]. However, these approaches often extract generic imaging characteristics that may lack specificity for EGFR mutation detection. Recent advances in artificial intelligence, particularly deep learning (DL) algorithms, offer a solution through their ability to learn discriminative features autonomously from imaging data [11, 12, 13]. Owing to their powerful feature‐extraction capabilities, DL models have demonstrated expert‐level performance across various fields, such as skin cancer classification [14], hematoma expansion prediction [15], and low‐grade and high‐grade gliomas prediction [16]. Promising results have also been observed in lung cancer research [17, 18]. Unlike conventional radiomic methods that rely on handcrafted features and require precise tumor segmentation, DL‐based radiomics can automatically learn clinically relevant representations, resulting in more accurate and outcome‐specific predictions.

We hypothesize that the EGFR genotype in lung adenocarcinoma is associated with specific imaging phenotypes and metabolic characteristics. To explore this hypothesis, we developed a DL model capable of extracting relevant PET/CT imaging features correlated with EGFR mutation status. In this study, we established and validated a comprehensive Clinical‐Radiomics‐Deep learning (CRD) model for predicting EGFR mutations in patients with lung adenocarcinoma. This model integrates radiomic features from PET/CT imaging with clinical data, and is further refined into a risk‐scoring tool using a nomogram‐based approach.

## 2 Materials and Methods

### 2.1 Patient Selection

**FIGURE 1:**
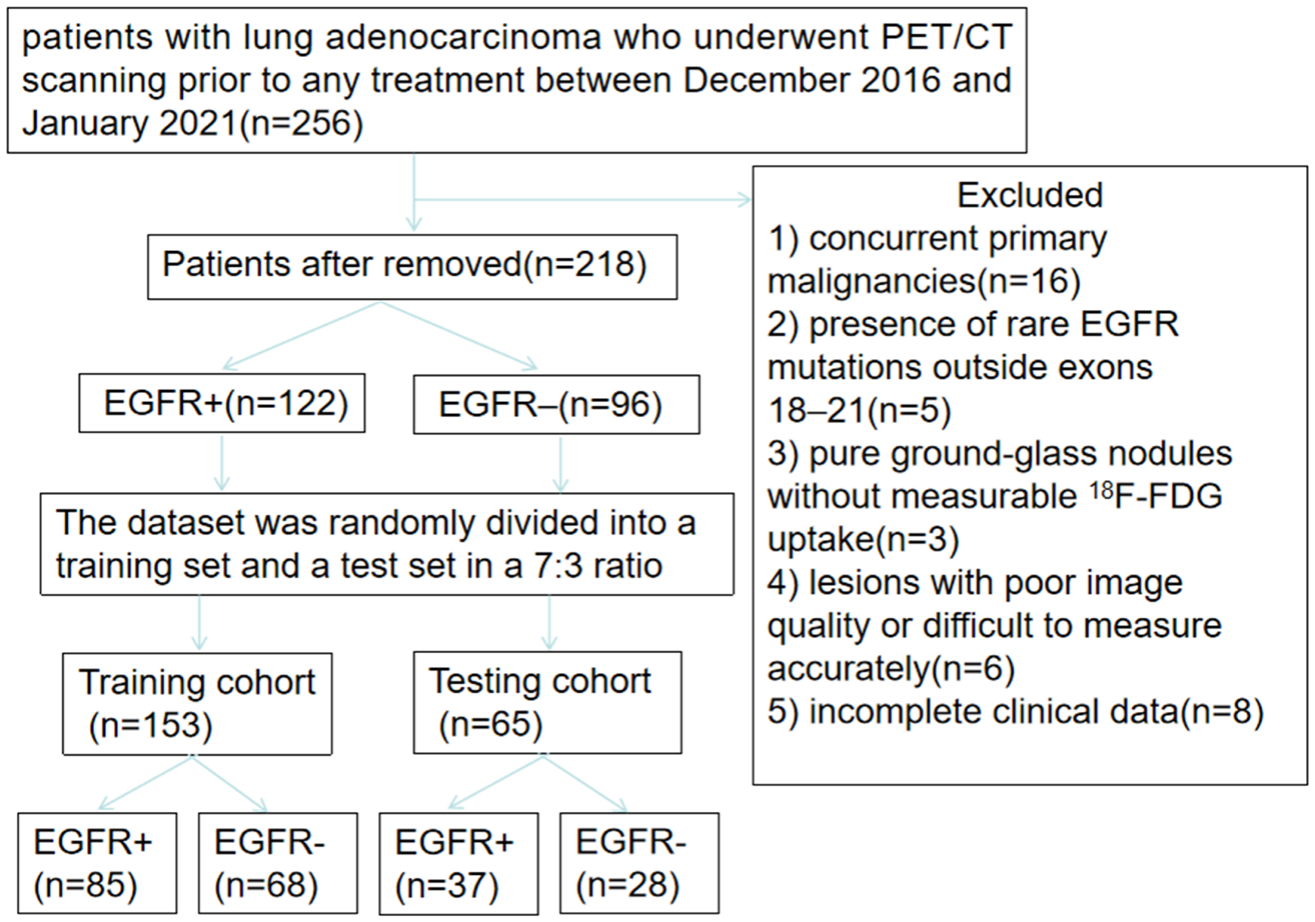
A flowchart of the patient selection process.

**FIGURE 2:**
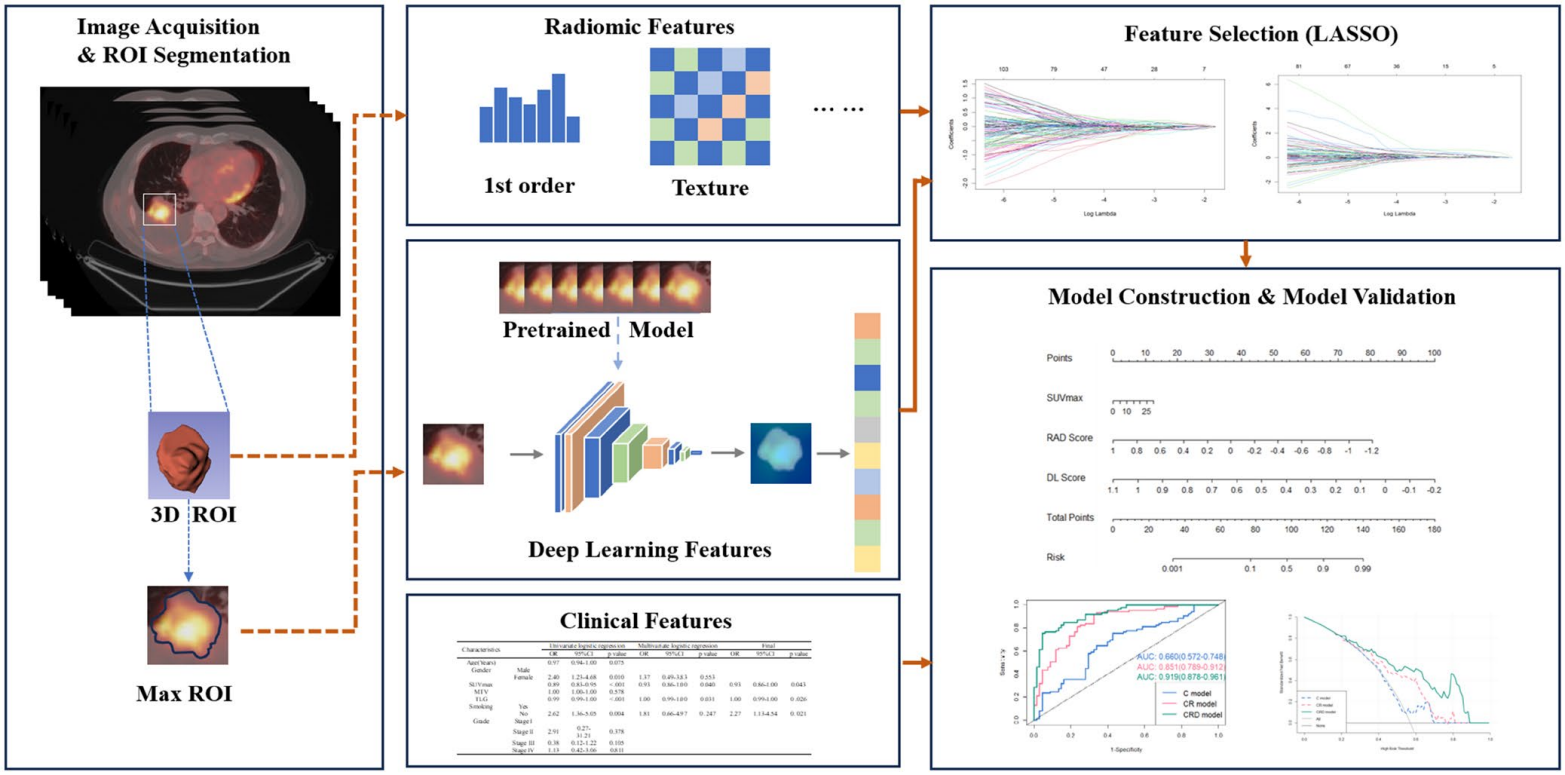
Workflow of the study.

A flowchart of the patient selection process is shown in Figure 1. The workflow of this study is illustrated in Figure 2. We retrospectively reviewed consecutive patients with histologically confirmed lung adenocarcinoma who underwent ^18^F‐FDG PET/CT scanning prior to any treatment between December 2016 and January 2021 at Zhejiang Cancer Hospital. All clinical variables, EGFR mutation status, and imaging data were complete for all patients, with no missing key variables. Therefore, no complex data imputation was necessary. Any patient who did not meet all inclusion criteria or met any of the exclusion criteria was excluded from the analysis, with the specific numbers detailed in Figure 1.

Inclusion criteria were as follows: (1)Pathological confirmation of lung adenocarcinoma via surgical resection or biopsy; (2)Availability of EGFR mutation status; (3) ^18^F‐FDG PET/CT imaging and EGFR mutation testing conducted within one month of each other; (4)No prior anti‐tumor therapy before PET/CT examination; (5)No history of other malignancies.

Exclusion criteria included: (1) Concurrent primary malignancies (*n* = 16); (2) Presence of rare EGFR mutations outside exons 18–21 (*n* = 5); (3) Pure ground‐glass nodules without measurable ^18^F‐FDG uptake (*n* = 3); (4) Lesions with poor image quality or difficult to measure accurately (*n* = 6) and (5) incomplete clinical data (*n* = 8).

### 2.2 EGFR Mutation Testing

EGFR mutation analysis was performed using histological samples obtained from surgical resections, employing a real‐time fluorescence PCR assay (Human EGFR Gene Mutation Detection Kit, AmoyDx, Xiamen, China). The results were interpreted in accordance with the manufacturer's instructions. Tumors harboring mutations in EGFR exons 18–21 were classified as EGFR‐mutant, whereas those without mutations were classified as EGFR wild‐type.

### 2.3 Image Acquisition

The PET/CT acquisition followed the Image Biomarker Standardization Initiative (IBSI) guidelines. All patients were scanned using the same PET/CT system (Discovery 710 PET/CT, GE Healthcare, Milwaukee, WI, USA) under standardized protocols. Patients fasted for 6–8 h prior to imaging to ensure blood glucose levels ≤ 11.1 mmol/L.

^18^F‐FDG was administered intravenously at a dose of 3.7 MBq/kg, and images were acquired 60 ± 5 min postinjection. Patients were instructed to empty their bladder and drink ≥ 500 mL of water before scanning.

The scan range extended from the skull base to the proximal thighs. Acquisition parameters included 2–3 min per bed position for the torso and a dedicated 8–10 min 3D acquisition for the cranial region. CT parameters were 120 kV, 100 mA, and 3 mm slice thickness. PET images were reconstructed using iterative reconstruction with CT‐based attenuation correction, producing whole‐body PET, CT, and fused PET/CT images, displayed on the MedEx system. The following metabolic parameters were calculated using vendor‐provided software: Maximum standardized uptake value (SUV_max_); Mean standardized uptake value (SUV_mean_); Metabolic tumor volume (MTV); Total lesion glycolysis (TLG), calculated as SUV_mean_ × MTV; MTV was measured using the adaptive threshold method. Volumes of interest (VOIs) were manually adjusted on axial, coronal, and sagittal planes to ensure full tumor coverage.

### 2.4 ROI Segmentation

VOIs were independently delineated by two experienced radiologists (YW and ZQC, > 10 years of experience) using 3D Slicer (version 5.6.2, https://www.slicer.org). Inter‐observer agreement was considered acceptable if the overlap coefficient exceeded 0.75; otherwise, a third senior radiologist conducted the segmentation.

PET and CT images were acquired simultaneously on the same scanner. Initial VOI delineation was performed on PET images and registered to corresponding CT images. A semiautomatic segmentation method with a 40% SUVmax threshold, based on MedEx workstation standards, was applied using the “PET Tumor Segmentation” tool, generating label files in NIfTI format for subsequent analysis.

### 2.5 Radiomic Feature Extraction

As illustrated in Figure 2, three‐dimensional tumor segmentation was performed using 3D Slicer, and radiomic features were subsequently extracted with the PyRadiomics Python package [19]. To ensure uniformity across datasets, all images were resampled to an isotropic voxel size of 1 × 1 × 1 mm^3^ prior to analysis.

Before feature extraction, a 3D wavelet transformation was applied to each image, generating eight sub‐band images (LLH, LHL, LHH, HLL, HLH, HHL, HHH, and LLL). From these images, a comprehensive set of radiomic features was extracted, including 14 shape features, 18 first‐order statistical features, 24 gray‐level co‐occurrence matrix (GLCM) features, 16 gray‐level size zone matrix (GLSZM) features, 16 gray‐level run length matrix (GLRLM) features, 14 gray‐level dependence matrix (GLDM) features, and five neighboring gray tone difference matrix (NGTDM) features. In total, 851 radiomic features were derived for each lesion.

### 2.6 Deep Learning Feature

In Two‐dimensional regions of interest (ROIs) from PET/CT images were used as inputs for deep learning‐based feature extraction. Five different neural network architectures were evaluated in this study: DenseNet169, ResNet50, ConvNext, Swin Transformer, and EfficientNet. All delineated ROI images were compiled into a dataset, which was used to train each of the five models independently. For each architecture, the set of model weights that achieved the highest classification accuracy during training was selected for further analysis.

The training and validation history of the deep learning network is shown in Figure S1. Following model training, the PET/CT image slice containing the largest ROI was selected for each lesion. This image was resized to 224 × 224 pixels to match the input requirements of the networks and then fed into the corresponding trained model. Feature vectors were extracted from the penultimate layer, that is, the layer immediately preceding the final classification output. The number of deep learning features extracted varied by network architecture, yielding 1664 features from DenseNet169, 2048 features from ResNet50, 1024 features each from ConvNext and Swin Transformer, and 1280 features from EfficientNet.

### 2.7 Clinical Feature Selection

Clinical variables including age, sex, smoking history, tumor stage, SUVmax, MTV, and TLG were collected. Univariate logistic regression was performed to identify variables significantly associated with EGFR mutation status (*p* < 0.05). These variables were then entered into a multivariate logistic regression with stepwise backward selection.

### 2.8 Radiomic and Deep Learning Feature Selection

Feature selection was conducted using the Least Absolute Shrinkage and Selection Operator (LASSO) regression, which applies L1 regularization to achieve model sparsity and reduce overfitting.

The selection process included the following steps: (1) *Z*‐score normalization of radiomic and deep learning features; (2) Two‐sided *t*‐tests to identify features with *p* ≤ 0.05; (3) LASSO regression with 10‐fold cross‐validation to determine the optimal penalty parameter (*λ*); (4) Selection of features corresponding to the model with the lowest mean squared error (MSE).

### 2.9 Cross‐Validation‐Based Feature Selection and Score Construction

In this study, the dataset was first divided into a training set and a test set, with the test set used independently for the final model performance evaluation. A five‐fold cross‐validation (5‐FCV) strategy was applied only within the training set to ensure the reliability and reproducibility of the model evaluation. In each iteration of the 5‐FCV, the training set was randomly split into five equally sized subsets: four subsets (approximately 80% of the total training set) served as the sub‐training set for model training and parameter optimization, while the remaining one subset (approximately 20% of the total training set) was used as the validation set for intermediate performance validation.

To select stable features, the feature selection methods described in Sections 2.7 and 2.8 were applied separately in each fold, and the frequency of feature selection was recorded for each type of feature. The threshold for feature selection was set to selected in at least three folds. For the radiomics and deep learning features selected, their regression coefficients from each fold's LASSO regression were extracted (with coefficients for unselected features assigned a value of 0), and the average of the fivefold coefficients was used as the final stable coefficient for each feature. This design effectively reduces the randomness bias introduced by a single data split and enhances the stability and reliability of the feature weights.

Based on the stable feature coefficients, the radiomics score and deep learning score for each case were calculated by performing a weighted sum of the respective radiomics and deep learning features. These two scores, along with the selected clinical features, together formed the core input features for subsequent model training.

### 2.10 Model Construction

Classification is a central focus in machine learning research, and in this study, five commonly used algorithms were employed for model development: logistic regression, decision tree, random forest, support vector machine (SVM), and XGBoost. The selected clinical, radiomic, and deep learning features were incorporated into these models to perform predictive analyses. The model performance was evaluated using 5‐FCV., and the combination of machine learning algorithm and feature set that yielded the highest performance was identified as the optimal configuration.

Based on this optimal machine learning model, a comprehensive clinical‐radiomics‐deep learning model (CRD model) was constructed. For comparative purposes, a clinical‐only model (C model) and a combined clinical‐radiomics model (CR model) were also developed. To enhance the clinical utility of the predictive framework, a nomogram was generated based on the CRD model, and a corresponding risk scoring system was established to facilitate individualized clinical decision‐making.

### 2.11 Statistical Analysis

Statistical analyses were performed using R (version 4.3.2, http://www.r‐project.org) and Python (version 3.8). Continuous variables were expressed as mean ± standard deviation ($\bar{x}$ ± s) or median (interquartile range) depending on the distribution. Student's *t*‐test was used for comparisons of continuous variables, while categorical variables were compared using the chi‐square test.

Univariate and multivariate logistic regression analyses were conducted using the autoReg R package. LASSO logistic regression was performed using the glmnet package. Model performance was evaluated based on area under the ROC curve (AUC), accuracy, sensitivity, specificity, positive predictive value (PPV), and negative predictive value (NPV). ROC curves were plotted using the pROC package. The DeLong test, implemented in the pROC package, was used to compare the AUCs of the models on the independent test set. Since multiple pairwise comparisons were conducted, the Holm–Bonferroni method was applied to adjust for multiple comparisons and control the type I error rate. A corrected *p*‐value < 0.05 was considered statistically significant. For the calibration curve, the calibrate() function from the rms package was used, with method = “boot” and *B* = 1000 to perform 1000 bootstrap resamples. The difference between the predicted probability and the actual event rate was calculated for each resample to obtain the calibration curve and its confidence intervals. For the Decision Curve Analysis curve, the decision_curve() function from the rmda package was used, with bootstraps = 100 to perform 100 bootstrap resamples, so as to evaluate the net benefit and its variability across different risk thresholds.

## 3 Results

### 3.1 Patient Clinical Characteristics

The detailed characteristics of all patients are summarized in Table 1. A total of 218 samples were included, comprising 122 patients with EGFR mutations and 96 with wild‐type EGFR. The dataset was randomly divided into a training set and a test set in a 7:3 ratio. Seven clinical variables potentially associated with EGFR mutation status were collected. By conducting a comprehensive evaluation of the univariate and multivariate logistic regression results from 5‐FCV (Table S1), we selected features based on predefined stability criteria. Ultimately, SUVmax was chosen as the clinical feature for model construction due to its stable performance in the analysis results.

**TABLE 1:** Clinical characteristics of all the patients.

Characteristics		Training cohort (*n* = 153)			Testing cohort (*n* = 65)		
		EGFR – (*n* = 68)	EGFR + (*n* = 85)	*p*	EGFR – (*n* = 28)	EGFR + (*n* = 37)	*p*
Age, year		63.50 ± 11.49	60.33 ± 10.19	0.077	63.04 ± 14.21	63.41 ± 10.02	0.907
Gender	Male	47 (69.12%)	41 (48.24%)	0.015	18 (64.29%)	12 (32.43%)	**0.021**
	Female	21 (30.88%)	44 (51.76%)		10 (35.71%)	25 (67.57%)	
SUV_max_		13.46;8.79–16.04	10.09;7.36–12.67	0.001	11.98; 9.35–15.03	10.45; 6.63–15.03	0.172
MTV		10.19; 3.80–26.13	5.43; 2.30–10.21	0.001	8.45; 5.20–19.25	8.56 5.56–12.52	0.740
TLG		90.25;26.27–232.48	29.97; 12.64–65.96	< 0.001	60.58; 32.56–119.53	46.59; 18.98–93.91	0.177
Smoking	Yes	40 (58.82%)	30 (35.29%)	0.006	14 (50.00%)	10 (27.03%)	0.101
	No	28 (41.18%)	55 (64.71%)		14 (50.00%)	27 (72.97%)	
Stage	Stage I	8 (11.76%)	11 (12.94%)	0.042	2 (7.14%)	4 (10.81%)	0.060
	Stage II	1 (1.47%)	4 (4.71%)		3 (10.71%)	0 (0.00%)	
	Stage III	21 (30.88%)	11 (12.94%)		9 (32.14%)	6 (16.22%)	
	Stage IV	38 (55.88%)	59 (69.41%)		14 (50.00%)	27 (72.97%)	

*Note:* Bold value is statistically significant.

### 3.2 Results of Feature Selection

A deep learning dataset was constructed based on the randomly assigned patient data described above. In total, the training set comprised 793 images from EGFR‐mutant cases and 760 images from EGFR wild‐type cases, while the test set included 370 and 291 images, respectively. All deep learning models were trained using consistent parameters, which are detailed in the Supporting Information (Table S2). The model weights yielding the highest accuracy were selected for subsequent feature extraction.

After feature extraction, this study first performed stable feature selection based on the cross‐validation method described in Section 2.9, and generated radiomics scores and deep learning scores. To further identify the optimal combination of feature sets and classifiers for improving model prediction performance, the study systematically evaluated the compatibility of different feature sets and machine learning classifiers through five‐fold cross‐validation within the training set.

During the combined evaluation process, classifier parameters were optimized via grid search on the sub‐training set of each fold, with performance on the sub‐validation set serving as the criterion for parameter selection. The specific parameters used in the grid search are provided in Table S3. The combination performance of feature sets and classifiers was evaluated by the average AUC of each fold's validation set in the five‐fold cross‐validation, with specific results shown in Table 2.

**TABLE 2:** Depicts the AUC values of different machine learning algorithms for different feature building models on the validation set.

	Cli	Rad	Con	Eff	Res	Swin	Den
LR	0.644	**0.848**	**0.890**	**0.764**	0.843	**0.766**	**0.741**
RF	0.717	0.788	0.832	0.747	0.761	0.639	0.683
SVM	0.729	0.848	0.889	0.772	0.843	**0.766**	0.739
DT	0.707	0.796	0.827	0.699	0.768	0.722	0.659
XGB	**0.761**	0.847	0.872	0.748	**0.844**	0.732	0.733

*Note:* The Ave at the horizontal head of the table represents the average level of performance of a certain machine learning algorithm on different features. The Ave at the vertical head of the table represents the average performance of a feature combined with different machine learning algorithms. Bold values are statistically significant.

Abbreviations: Cli, clinical feature; Con, ConvNext feature; Den, DenseNet169 feature; DT, decision tree; Eff, EfficientNet feature; LR, logistic regression; Rad, radiomic feature; Res, ResNet50 feature; RF, random forest; SVM, support vector machine; Swin, Swin Transformer feature; XGB, xgboost.

In terms of classifier performance, the logistic regression (Logistic Regression) classifier outperformed all other classifiers across all feature sets, with its AUC values higher than those of other classifiers in all five feature sets; the xgboost classifier performed second, making it the second‐best choice. Regarding feature set performance, the ConvNext feature demonstrated strong predictive power, particularly in combination with logistic regression, where the AUC reached 0.890, the highest value among all feature set‐classifier combinations. Based on these performance evaluation results, the combination of ConvNext features and logistic regression was selected as the core framework for subsequent model construction.

### 3.3 Performance Comparison of the Three Models

**FIGURE 3:**
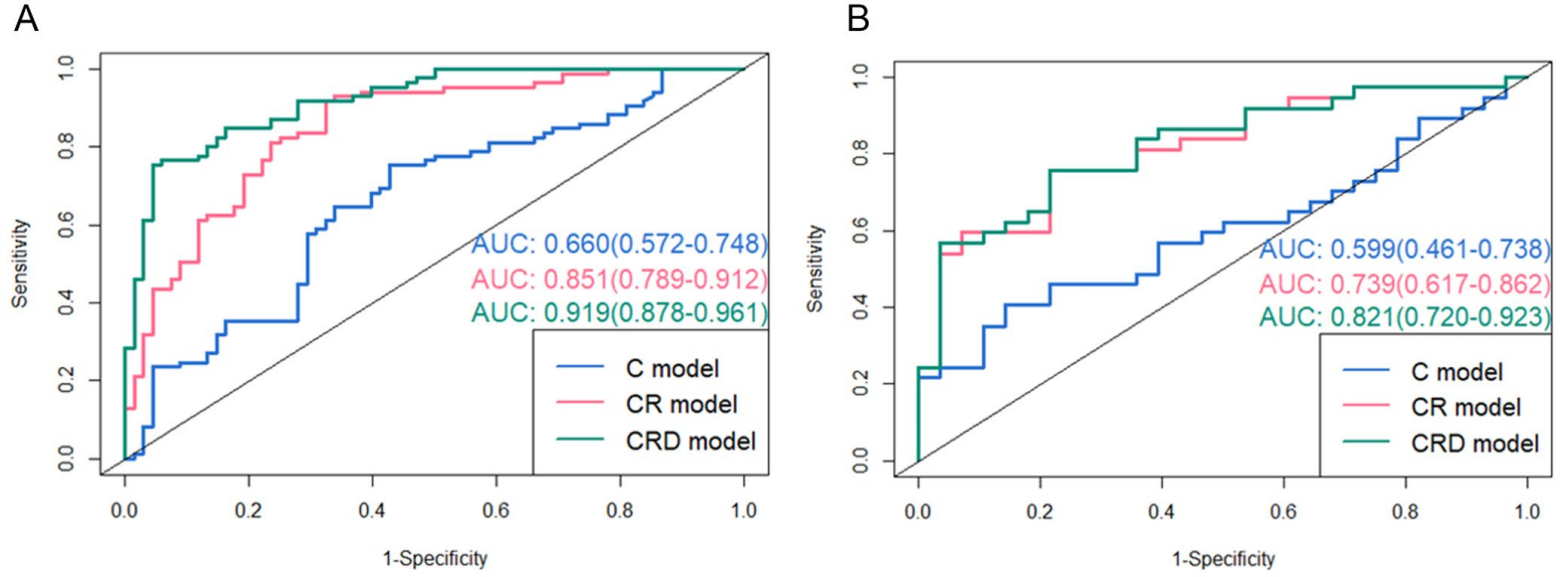
Receiver operating characteristics curves of the three models for the (A) training and (B) test cohorts, respectively. The training cohort (A) includes *n* = 153 patients, and the test cohort (B) includes *n* = 65 patients. C model, clinical model; CR model, clinical‐radiomics model; CRD model, clinical‐radiomics‐deep learning model. Values in parentheses are 95% confidence intervals (95% CIs) of the area under the ROC curve (AUC).

We re‐trained the model on the entire training set using the selected optimal classifier. The predictive performance of the three models on both the training and test sets is summarized in Table 3, with corresponding ROC curves presented in Figure 3. The DeLong test was employed to compare the ROC curves on the test set. A statistically significant difference was observed between the CRD model and the C model (*Z* = −3.522, *p* < 0.001, corrected *p* = 0.001), as well as between the CRD model and the CR model (*Z* = −2.197, *p* = 0.028, corrected *p* = 0.028).

**TABLE 3:** The performance of the clinical model, clinical‐radiomics model and clinical‐radiomics‐deep learning model.

	AUC (95% CI)	ACC (95% CI)	SEN	SPE	PPV	NPV
Train cohort						
C model	0.660 (0.572–0.748)	0.627 (0.510–0.745)	0.776	0.441	0.635	0.612
CR model	0.851 (0.789–0.912)	0.764 (0.662–0.868)	0.835	0.676	0.763	0.767
CRD model	0.919 (0.878–0.961)	0.830 (0.739–0.921)	0.847	0.809	0.847	0.809
Test cohort						
C model	0.599 (0.461–0.738)	0.523 (0.402–0.644)	0.649	0.357	0.571	0.435
CR model	0.739 (0.617–0.862)	0.692 (0.580–0.805)	0.757	0.607	0.718	0.654
CRD model	0.821 (0.720–0.923)	0.754 (0.650–0.859)	0.757	0.750	0.800	0.700

Abbreviations: ACC, accuracy; AUC, area under the curve; C model, clinical model; CI, confidence interval; CR model, clinical‐radiomics model; CRD model, clinical‐radiomics‐deep learning model; NPV, negative predictive value; PPV, positive predictive value; SEN, sensitivity; SPE, specificity.

These results indicate that the CRD model demonstrated superior predictive performance for EGFR mutation risk. Specifically, in the test set, the CRD model achieved an AUC of 0.821, which was significantly higher than that of the C model (AUC = 0.599) and the CR model (AUC = 0.737).

To validate the performance of the CRD model, an ablation study was conducted, and the results are presented in Table 4. The CRD model achieved an AUC of 0.821 (95% CI: 0.720–0.923), which is comparable to the best‐performing CD model (AUC = 0.821, 95% CI: 0.718–0.921) and significantly higher than the other models (CR model AUC = 0.739, RD model AUC = 0.815).

**TABLE 4:** The performance of the radiomics‐deep learning model, clinical‐radiomics model, clinical‐deep learning model and clinical‐radiomics‐deep learning model.

	AUC (95% CI)	ACC (95% CI)	SEN	SPE	PPV	NPV
RD model	0.815 (0.711–0.918)	0.723 (0.614–0.823)	0.757	0.679	0.757	0.679
CR model	0.739 (0.617–0.862)	0.692 (0.580–0.805)	0.757	0.607	0.718	0.654
CD model	0.821 (0.718–0.921)	0.738 (0.631–0.845)	0.730	0.750	0.794	0.677
CRD model	0.821 (0.720–0.923)	0.754 (0.650–0.859)	0.757	0.750	0.800	0.700

Further evaluation showed that the CRD model demonstrated balanced and relatively high values across four key metrics: sensitivity (SEN, 0.757), specificity (SPE, 0.750), positive predictive value (PPV, 0.800), and negative predictive value (NPV, 0.700). Its SEN was on par with the RD and CR models, while outperforming the CD model, ensuring a lower risk of missed diagnoses. The SPE was comparable to the CD model and significantly better than the RD and CR models, which helps to reduce the likelihood of misdiagnosis. Both PPV and NPV were the highest among all the compared models, further enhancing the clinical reliability of the model's predictions.

**FIGURE 4:**
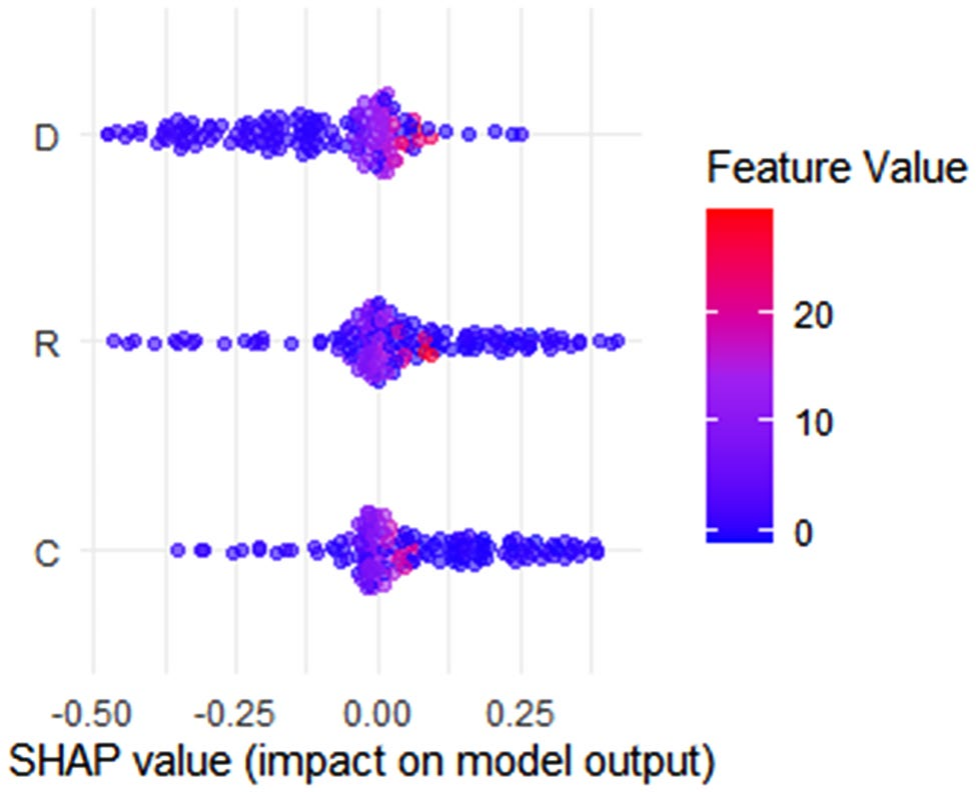
SHAP analysis: features C, R, D on CRD model output and feature value correlation.

We utilized the SHAP method to visualize the impact of three features of CRD on the model output (Figure 4). Each horizontal point represents a data point's SHAP value, reflecting the contribution of the corresponding feature to the model's prediction. The color of the points indicates the size of the feature value, with blue representing lower values and red representing higher values. For the D feature, points with higher feature values tend to cluster in the positive SHAP value region, indicating that higher feature values are more likely to promote the model's prediction. For the R feature, SHAP values are concentrated around zero, with contributions from high and low feature values being relatively balanced. For the C feature, the SHAP values are more dispersed, suggesting that the impact of this feature on the model's prediction varies across different feature values.

**FIGURE 5:**
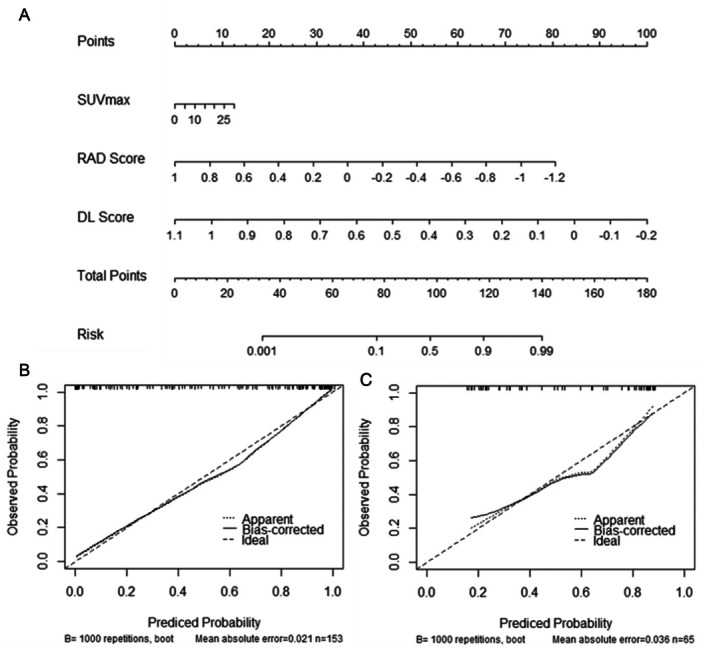
CRD nomogram and its calibration curves. (A) Nomogram of the CRD model for predicting EGFR mutation in the train cohort. (B) Calibration curves for the CRD nomogram on the train cohort. (C) Calibration curves for the CRD Nomogram on the test cohort. Calibration curves indicate the goodness‐of‐fit of the nomogram. The 45° straight line represents the perfect match between the actual (*Y*‐axis) and Nomogram‐predicted (*X*‐axis) probabilities. As the distance between the two curves decreases, the accuracy increases.

Based on the training set data, we constructed a nomogram by integrating radiomic features, deep learning‐derived feature scores, and clinical variables using logistic regression, as shown in Figure 5A. The calibration curves of the nomogram are presented in Figure 5B,C, which were used to assess the agreement between predicted probabilities and actual outcomes.

**FIGURE 6:**
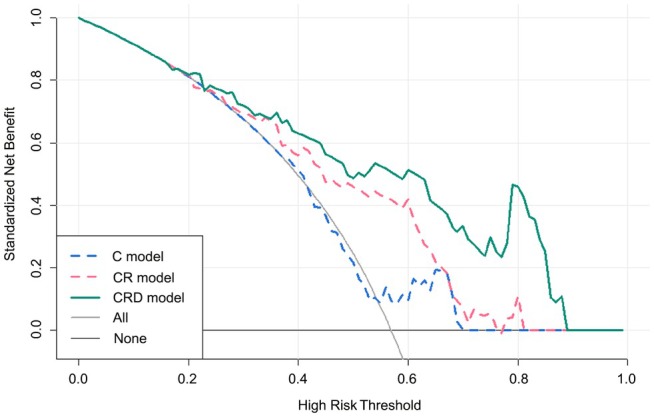
Decision curve analysis on the independent test set. The *y*‐axis indicates the net benefit and the *x*‐axis indicates threshold probability. Net benefit is plotted as a function of threshold probability for the C (clinical), CR (clinical‐radiomics), and CRD (clinical‐radiomics‐deep learning) models. The gray and black lines represent the All (treat all) and None (treat none) strategies, respectively. The CRD model demonstrated the highest net benefit across a wide range of thresholds, indicating its superior clinical utility.

To evaluate the clinical utility of the model, decision curve analysis (DCA) was performed, as illustrated in Figure 6. The DCA results indicated that the CRD model provided greater overall net benefit across a range of threshold probabilities compared to the other two models. These findings suggest that the nomogram is well calibrated and holds potential clinical value.

## 4 Discussion

In this study, we employed machine learning algorithms to integrate clinical, radiomic, and deep learning features, aiming to assess the value of PET/CT imaging in predicting EGFR mutation status. The developed CRD model demonstrated robust predictive performance, achieving an AUC of 0.821 on the test set, thereby highlighting the advantage of multi‐omics feature fusion for EGFR mutation prediction. This approach offers a noninvasive method for EGFR status assessment and has the potential to complement traditional biopsy‐based methods.

EGFR mutations are major oncogenic drivers in NSCLC and are predictive biomarkers for the efficacy of FDA‐ and CFDA‐approved EGFR TKIs. Patients with EGFR‐mutant NSCLC tend to be female, nonsmokers, and have adenocarcinoma histology [7, 8]. Our findings are consistent with these patterns, as sex and smoking history were significantly associated with EGFR mutation status, while age and TNM stage were not. Previous studies have combined clinical factors with radiomics to predict EGFR mutations [20, 21]. For instance, nomograms integrating clinical features such as sex, nonsmoking status, SUVmean, MTV, and pleural retraction have shown predictive utility. One study using PET/CT imaging and clinical data from 274 lung adenocarcinoma patients achieved an AUC of 0.805 [22]. Although clinical models (C models) are intuitive and interpretable, they reflect only limited pathological‐level tumor information.

Radiomics, on the other hand, captures quantitative features from PET/CT images, offering macro‐level insight into tumor heterogeneity and genotype correlation. Studies such as those by Gao et al. [23], Zhao et al. [24], Li et al. [25], Zhang et al. [26], Liu et al. [27], and Chang et al. [28] have all demonstrated that combining radiomic features with clinical data improves EGFR mutation prediction. However, radiomic methods require labor‐intensive tumor segmentation and may lack genotype specificity.

To overcome these limitations, we introduced deep learning models capable of automatically learning EGFR‐related tumor characteristics, eliminating the need for manual segmentation. Unlike traditional radiomics, which involves multiple complex steps (segmentation, extraction, selection, modeling), our deep learning approach only requires a predefined tumor ROI. Moreover, the deep learning model captures both the tumor and its surrounding microenvironment, such as pleural traction, and is more efficient and user‐friendly.

While traditional radiomics focuses on low‐ and mid‐level image features, deep learning excels at capturing abstract, high‐dimensional features essential for EGFR prediction. Wang et al. [29] demonstrated that deep learning significantly outperforms handcrafted CT or clinical features (AUC = 0.81). Wang et al. [30] further validated deep learning's utility using CT and gene sequencing data from 18,232 patients across nine cohorts, with FAIS models achieving AUCs of 0.748–0.813.

Yoon et al. [31] and Yin et al. [32] also highlighted the superiority of deep learning models in specific subgroups and modalities. Zhao et al. [33] reported a 3D CNN model that achieved AUCs of 75.8% and 75.0% on internal and public datasets, respectively. Denseformer [34] and EME [35] models further confirmed deep learning's superior performance, with EME reaching an AUC of 0.907, and the fusion model attaining 0.941. Our CRD model achieved AUCs of 0.919 (training set) and 0.821 (test set), reinforcing its predictive strength.

The advantages of our deep learning model include: Automatic feature extraction from hierarchical representations directly correlated with EGFR status; No requirement for manual tumor delineation, thus reducing workload and variability; Integration of the tumor microenvironment, potentially enhancing predictive accuracy; Clinical usability, requiring only PET/CT input without additional preprocessing. Clinically, the model holds promise in multiple scenarios: As a noninvasive predictor of EGFR mutation status; As a complementary validation tool when biopsy results are wild‐type, to address potential sampling bias due to intratumoral heterogeneity.

This study adopted a 2D largest‐slice‐based deep learning feature extraction strategy. This choice was a trade‐off made after considering the current dataset size, computational efficiency, and the need for fair comparison among multiple architectures. Although this method cannot capture the 3D spatial context of lesions, it offers better computational efficiency and anti‐overfitting capability. Additionally, the selection criterion of the largest slice is objective and reproducible, which has been proven effective in numerous studies [36]. Future work will focus on developing and applying 3D deep learning models based on a larger sample size.

Limitations of this study include: Population bias—patients were exclusively from Asian cohorts, limiting generalizability across ethnicities; Retrospective design—necessitating prospective validation with larger, multicenter datasets; Narrow focus on EGFR mutations—future work could extend to ALK, ROS‐1, and other genetic alterations; Barriers to deployment—including variations in imaging equipment, lack of algorithm transparency, and the “black box” nature of deep learning, which impedes interpretability.

Our decision to exclude rare EGFR mutations outside exons 18–21 was primarily driven by methodological and study objective considerations:

Ensuring Group Homogeneity: Common EGFR mutations (e.g., Exon 19 del, L858R) and rare mutations can differ significantly in incidence, response to TKIs, and potentially biology. Combining them into a single “mutant” group could introduce noise, making it difficult for the model to learn stable and consistent imaging signatures, thereby compromising its core discriminative power.

Primary Study Aim: The primary objective of this study was to build a model that reliably distinguishes “Common EGFR Mutant” from “Wild‐Type” status. This addresses a fundamental and highly clinically relevant question. Incorporating or separately analyzing rare mutations (typically with a prevalence of < 5%) would require a larger sample size and a different modeling strategy (e.g., a multi‐class model), which was beyond the scope of this initial validation study.

Impact on Real‐World Applicability and Future Plans: We fully agree that an ideal clinical tool should ultimately handle all mutation types. Therefore, we have made the detection and classification of rare mutations one of our highest priorities for future work. As our dataset expands, we plan to develop an advanced model capable of differentiating between “common mutant,” “rare mutant,” and “wild‐type” categories. At this stage, for patients with a model‐predicted high risk (i.e., predicted mutant) but initial biopsy showing wild‐type, we would suggest heightened suspicion for a rare mutation and recommend more comprehensive genetic testing.

To enhance clinical applicability, future research should aim to standardize data acquisition, expand datasets across institutions, and incorporate explainable AI methodologies, thereby advancing the translational potential of the CRD model. We plan to collaborate with multiple medical centers, both domestic and international, to collect data involving diverse ethnicities and different PET/CT scanner models for external validation. We are designing a prospective clinical trial to further evaluate the predictive performance and clinical impact of our model for predicting EGFR mutation status in real‐world clinical workflows. To establish robust clinical decision thresholds, we further need to design prospective, multicenter studies. These studies will systematically integrate the net benefit data from DCA with cost‐effectiveness evaluation results, and we will invite frontline clinicians, patient representatives, and health policymakers to jointly participate in the deliberation of thresholds. Ultimately, we will define acceptable ranges of missed diagnosis risk and corresponding decision thresholds that are suitable for different clinical scenarios, so as to ensure their clinical applicability and ethical validity. In addition, External Validation and Collaboration: We are actively seeking collaboration with international partners who have access to diverse patient cohorts (including Caucasian, African descent, etc.) to obtain independent external datasets. This will allow us to validate the performance of our current model and critically assess its cross‐ethnic generalizability. Developing a More Generalizable Model: Should a performance drop be observed, we will explore strategies such as: Model Fine‐tuning: Adapting our pre‐trained model using a small amount of multi‐ethnic data. De Novo Training: Ultimately, integrating multicenter, multi‐ethnic data from the outset to develop a truly global predictive model. To address this, we have made temporal external validation a cornerstone of our future work. We also plan to collect new patient data from our institution from beyond 2021 (the end date of this study) to build a true temporal validation cohort to assess the long‐term robustness of our model. We fully acknowledge that a more ideal, albeit more time‐consuming, approach would be dual‐modality independent segmentation. This would help explore the optimal CT features and is a key direction for our future methodological refinements. We explicitly commit to incorporating ComBat harmonization (or similar methods) as a mandatory step in the preprocessing pipeline for our subsequent multicenter external validation. This is essential to ensure feature comparability across institutions and to build a truly robust and generalizable model. We have designated head‐to‐head comparison with established models as a key objective for our future external validation studies. Upon finalizing our model and acquiring multicenter collaboration data, we will proactively implement highly‐cited published models for a fair and direct performance evaluation on identical datasets.

## 5 Conclusions

This study demonstrates that a deep learning model based on ^18^F‐FDG PET/CT imaging holds significant potential for predicting EGFR mutation status in patients with lung adenocarcinoma. The integrated CRD model, which combines clinical data, radiomic features, and deep learning‐derived features, outperformed both the clinical‐only (C) model and the clinical‐radiomics (CR) model in predictive performance. Additionally, the developed nomogram provides a practical and intuitive tool for individualized EGFR mutation risk estimation, streamlining and enhancing the prediction process.

To our knowledge, this is one of the few studies to comprehensively integrate deep learning‐derived features from PET/CT with radiomics and clinical information for EGFR mutation prediction. Compared with traditional radiomics models, our deep learning framework captures high‐level abstract features directly related to EGFR genotype, offering a more efficient and generalizable approach.

These findings underscore the value of multi‐modal data integration in improving noninvasive diagnostic accuracy. Nonetheless, validation in larger, more diverse cohorts and prospective multicenter studies is essential to confirm the generalizability and clinical utility of this approach. Overall, the CRD model offers a promising supplementary tool for guiding targeted therapy decisions and advancing precision medicine in lung adenocarcinoma.

## Author Contributions

**Yun Wang:** methodology, formal analysis, investigation, data curation, writing – original draft, writing – review and editing, funding acquisition. **Zhaoqing Chen:** methodology, formal analysis, data curation, investigation, writing – review and editing. **Jing Li:** data curation. **Yuhuang Cai:** data curation, writing – review and editing. **Chengyang Sun:** data curation, writing – review and editing. **Jingjing Zhang:** writing – review and editing. **Marcus Hacker:** writing – review and editing. **Xiang Li:** conceptualization, methodology, writing – review and editing, supervision, project administration. **Heqing Yi:** conceptualization, methodology, data curation, writing – original draft, writing – review and editing, supervision, project administration, funding acquisition.

## Ethics Statement

This study was conducted in accordance with the Declaration of Helsinki and approved by the Ethics Committee of Zhejiang Cancer Hospital (IRB‐2023‐863).

## Consent

The Ethics Committee of Zhejiang Cancer Hospital waived the requirement for individual written informed consent for this retrospective analysis.

## Conflicts of Interest

The authors declare no conflicts of interest.

## Supporting information


**Table S1:–S3.**cam471370‐sup‐0001‐TablesS1–S3.docx.

**Figure S1:–S7.**cam471370‐sup‐0002‐FiguresS1–S7.docx.

**Text S1:–S6.**cam471370‐sup‐0003‐SupplementaryText.docx.

## Data Availability

The data may be available from the corresponding author based on reasonable request.
