# Supplementary material for: An Integrated Clinical‐Radiomics‐Deep Learning Model Based on 18F‐FDG PET/CT for Predicting EGFR Mutation Status in Lung Adenocarcinoma
Source: Cancer Med. 2025 Nov 20;14(22):e71370. doi: 10.1002/cam4.71370 (PMC12631541; doi:10.1002/cam4.71370)
Supplement: Supplementary file 1 — Table S1:–S3.cam471370‐sup‐0001‐TablesS1–S3.docx. [file CAM4-14-e71370-s003.docx]

**Supplementary Table 1.** Clinical characteristics selection result

|  | Characteristics |  | Univariate logistic regression | | | Multivariate logistic regression | | | Final | | |
| --- | --- | --- | --- | --- | --- | --- | --- | --- | --- | --- | --- |
|  |  |  | OR | 95%CI | *p* | OR | 95%CI | p value | OR | 95%CI | *p* |
| 1 | Age (Years) |  | 0.98 | 0.94-1.00 | 0.168 |  |  |  |  |  |  |
|  | Gender | Male |  |  |  |  |  |  |  |  |  |
|  |  | Female | 2.17 | 1.04-4.52 | 0.038 | 0.96 | 0.30-3.12 | 0.947 |  |  |  |
|  | **SUVmax** |  | 0.88 | 0.82-0.95 | <.001 | 0.91 | 0.84-0.98 | 0.020 | 0.91 | 0.84-0.98 | 0.019 |
|  | MTV |  | 1.00 | 1.00-1.00 | 0.610 |  |  |  |  |  |  |
|  | TLG |  | 1.00 | 0.99-1.00 | 0.004 | 1.00 | 0.99-1.00 | 0.138 | 1.00 | 0.99-1.00 | 0.136 |
|  | **Smoking** | Yes |  |  |  |  |  |  |  |  |  |
|  |  | No | 2.78 | 1.35-5.71 | 0.005 | 2.58 | 0.83-8.07 | 0.102 | 2.51 | 1.17-5.39 | 0. 018 |
|  | Stage | Stage Ⅰ |  |  |  |  |  |  |  |  |  |
|  |  | Stage Ⅱ | 3.50 | 0.31-39.15 | 0.309 |  |  |  |  |  |  |
|  |  | Stage Ⅲ | 0.46 | 0.13-1.69 | 0.245 |  |  |  |  |  |  |
|  |  | Stage Ⅳ | 1.44 | 0.48-4.36 | 0.519 |  |  |  |  |  |  |
| 2 | Age (Years) |  | 0.97 | 0.94-1.01 | 0.118 |  |  |  |  |  |  |
|  | Gender | Male |  |  |  |  |  |  |  |  |  |
|  |  | Female | 2.76 | 1.30-5.88 | 0.008 | 1.51 | 0.52-4.42 | 0.452 |  |  |  |
|  | SUVmax |  | 0.91 | 0.84-0.98 | 0.013 | 1.00 | 0.90-1.11 | 0.999 |  |  |  |
|  | MTV |  | 0.97 | 0.94-0.99 | 0.010 | 1.05 | 0.97-1.14 | 0.257 |  |  |  |
|  | **TLG** |  | 0.99 | 0.99-1.00 | 0.001 | 0.99 | 0.98-1.00 | 0.083 | 0.99 | 0.99-1.00 | 0.004 |
|  | **Smoking** | Yes |  |  |  |  |  |  |  |  |  |
|  |  | No | 2.92 | 1.38-6.17 | 0.005 | 1.55 | 0.53-4.55 | 0.427 | 2.30 | 1.05-5.07 | 0.038 |
|  | Stage | Stage Ⅰ |  |  |  |  |  |  |  |  |  |
|  |  | Stage Ⅱ | 4.21 | 0.27-43.12 | 0.578 |  |  |  |  |  |  |
|  |  | Stage Ⅲ | 0.32 | 0.09-1.20 | 0.092 |  |  |  |  |  |  |
|  |  | Stage Ⅳ | 1.29 | 0.43-3.83 | 0.650 |  |  |  |  |  |  |
| 3 | Age (Years) |  | 0.98 | 0.95-1.02 | 0.269 |  |  |  |  |  |  |
|  | Gender | Male |  |  |  |  |  |  |  |  |  |
|  |  | Female | 1.57 | 0.73-3.34 | 0.245 |  |  |  |  |  |  |
|  | SUVmax |  | 0.91 | 0.84-0.98 | 0.019 | 0.95 | 0.87-1.03 | 0.224 |  |  |  |
|  | MTV |  | 1.00 | 1.00-1.00 | 0.606 |  |  |  |  |  |  |
|  | **TLG** |  | 0.99 | 0.99-1.00 | 0.001 | 0.99 | 0.99-1.00 | 0.023 | 0.99 | 0.99-1.00 | 0.004 |
|  | Smoking | Yes |  |  |  |  |  |  |  |  |  |
|  |  | No | 2.27 | 1.05-4.90 | 0.036 | 2.12 | 0.94-4.77 | 0.070 | 2.09 | 0.93-4.68 | 0.072 |
|  | Stage | Stage Ⅰ |  |  |  |  |  |  |  |  |  |
|  |  | Stage Ⅱ | 1.87 | 0.15-23.40 | 0.625 |  |  |  |  |  |  |
|  |  | Stage Ⅲ | 0.38 | 0.09-1.51 | 0.167 |  |  |  |  |  |  |
|  |  | Stage Ⅳ | 1.11 | 0.33-3.73 | 0.871 |  |  |  |  |  |  |
| 4 | Age (Years) |  | 0.97 | 0.94-1.00 | 0.052 |  |  |  |  |  |  |
|  | **Gender** | Male |  |  |  |  |  |  |  |  |  |
|  |  | Female | 2.69 | 1.30-5.56 | 0.008 | 1.89 | 0.62-5.79 | 0.263 | 2.34 | 1.08-5.06 | 0.031 |
|  | **SUVmax** |  | 0.89 | 0.82-0.95 | 0.001 | 0.92 | 0.85-1.00 | 0.039 | 0.92 | 0.85-0.99 | 0.036 |
|  | MTV |  | 1.00 | 1.00-1.00 | 0.575 |  |  |  |  |  |  |
|  | TLG |  | 1.00 | 0.99-1.00 | 0.002 | 1.00 | 0.99-1.00 | 0.081 | 1.00 | 0.99-1.00 | 0.083 |
|  | Smoking | Yes |  |  |  |  |  |  |  |  |  |
|  |  | No | 2.47 | 1.21-5.02 | 0.013 | 1.33 | 0.45-3.97 | 0.608 |  |  |  |
|  | Stage | Stage Ⅰ |  |  |  |  |  |  |  |  |  |
|  |  | Stage Ⅱ | 1.78 | 0.13-23.52 | 0.662 |  |  |  |  |  |  |
|  |  | Stage Ⅲ | 0.49 | 0.14-1.68 | 0.260 |  |  |  |  |  |  |
|  |  | Stage Ⅳ | 1.23 | 0.43-3.51 | 0.701 |  |  |  |  |  |  |
| 5 | Age (Years) |  | 0.97 | 0.93-1.00 | 0.053 |  |  |  |  |  |  |
|  | **Gender** | Male |  |  |  |  |  |  |  |  |  |
|  |  | Female | 3.11 | 1.43-6.76 | 0.004 | 2.56 | 0.75-8.77 | 0.135 | 3.24 | 1.39-7.55 | 0.006 |
|  | **SUVmax** |  | 0.87 | 0.80-0.94 | <.001 | 0.89 | 0.81-0.97 | 0.009 | 0.89 | 0.81-0.97 | 0.008 |
|  | MTV |  | 1.00 | 1.00-1.00 | 0.594 |  |  |  |  |  |  |
|  | TLG |  | 1.00 | 1.00-1.00 | 0.003 | 1.00 | 0.99-1.00 | 0.104 | 1.00 | 0.99-1.00 | 0.111 |
|  | Smoking | Yes |  |  |  |  |  |  |  |  |  |
|  |  | No | 2.69 | 1.29-5.64 | 0.009 | 1.36 | 0.42-4.38 | 0. 607 |  |  |  |
|  | Stage | Stage Ⅰ |  |  |  |  |  |  |  |  |  |
|  |  | Stage Ⅱ | 1.50 | 0.12-18.36 | 0.751 |  |  |  |  |  |  |
|  |  | Stage Ⅲ | 0.26 | 0.07-1.01 | 0.052 |  |  |  |  |  |  |
|  |  | Stage Ⅳ | 0.69 | 0.21-2.21 | 0.529 |  |  |  |  |  |  |

**Supplementary Table 2.** The parameter settings of Deep Learning model.

| Setting item | value |
| --- | --- |
| Learning rate | 0.0001 |
| optimize | AdamW |
| loss function | CrossEntropyLoss |
| epochs | 100 |
| batch size | 32 |
| Learning rate Decay | Cosine Annealing |
| Droupout in fully connected layer | 0.5 |

**Supplementary Table 3.** Search Range of Hyperparameters for Machine Learning Models

| Model | R package/function | R Parameter Name | Grid Search Range |
| --- | --- | --- | --- |
| LR | rms::lrm | Default parameter | default |
| RF | randomForest::randomForest | mtry | 1,2,3 |
|  |  | ntree | 100,300,500 |
|  |  | nodesize | 1,3,5 |
| SVM | e1071::svm | cost | 0.1,1,10,100 |
|  |  | gamma | 0.01,0.1,1 |
| DT | rpart::rpart | cp | 0.001,0.01,0.1 |
|  |  | maxdepth | 3,5,7 |
| XGB | xgboost::xgboost | max_depth | 3,5,7 |
|  |  | eta | 0.01,0.1,0.3 |
|  |  | nrounds | 50,100,200 |
|  |  | gamma | 0,0.1,0.2 |
|  |  | min_child_weight | 3,5,7 |
