# Supplementary material for: An Integrated Clinical‐Radiomics‐Deep Learning Model Based on 18F‐FDG PET/CT for Predicting EGFR Mutation Status in Lung Adenocarcinoma
Source: Cancer Med. 2025 Nov 20;14(22):e71370. doi: 10.1002/cam4.71370 (PMC12631541; doi:10.1002/cam4.71370)
Supplement: Supplementary file 2 — Figure S1:–S7.cam471370‐sup‐0002‐FiguresS1–S7.docx. [file CAM4-14-e71370-s001.docx]

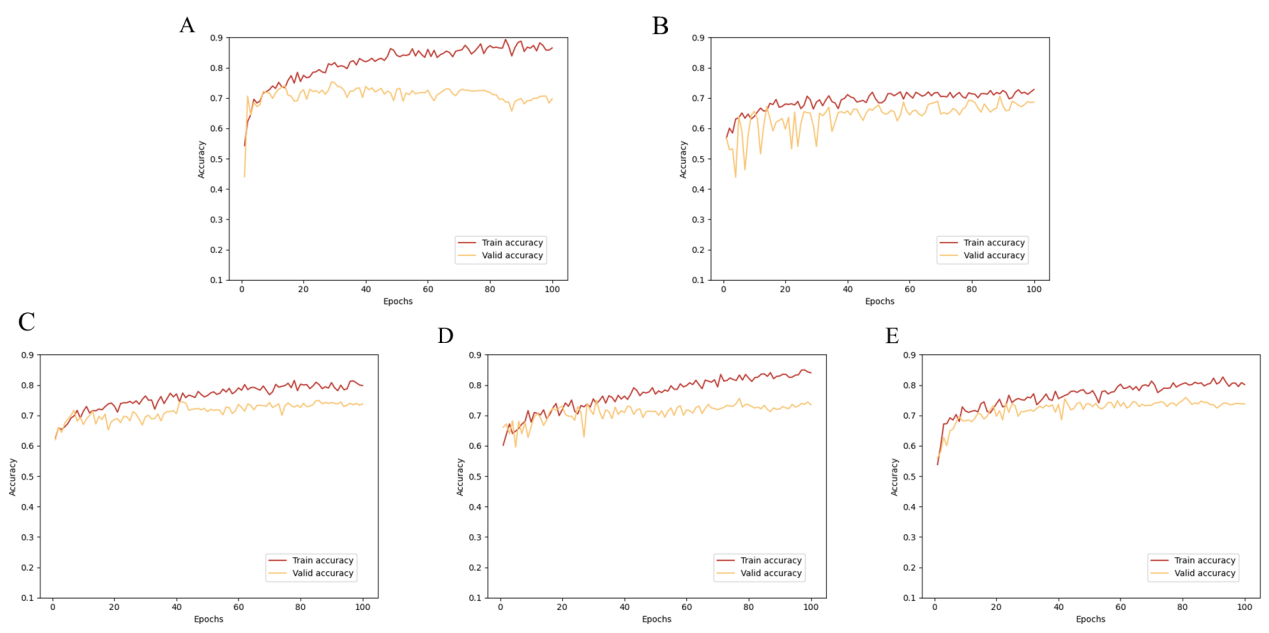


**Supplementary Figure 1.** The training and validation history of the deep learning network. A is Convnext, B is EfficientNet, C is ResNet50, D is Swin Transformer, E is DenseNet169.


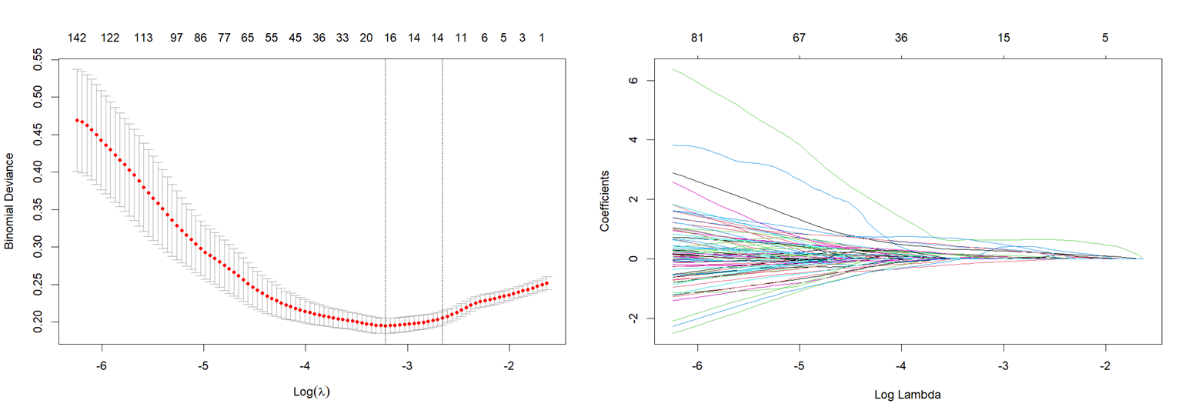


**Supplementary Figure 2.** Calculation of the LASSO logistic regression algorithm in Radiomic Feature.


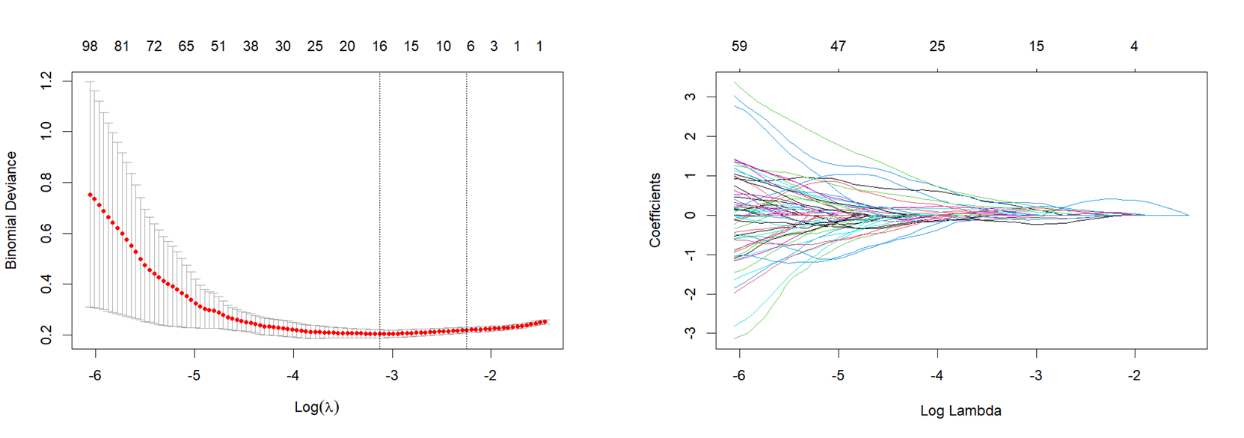


**Supplementary Figure 3.** Calculation of the LASSO logistic regression algorithm in Dense169 Feature.


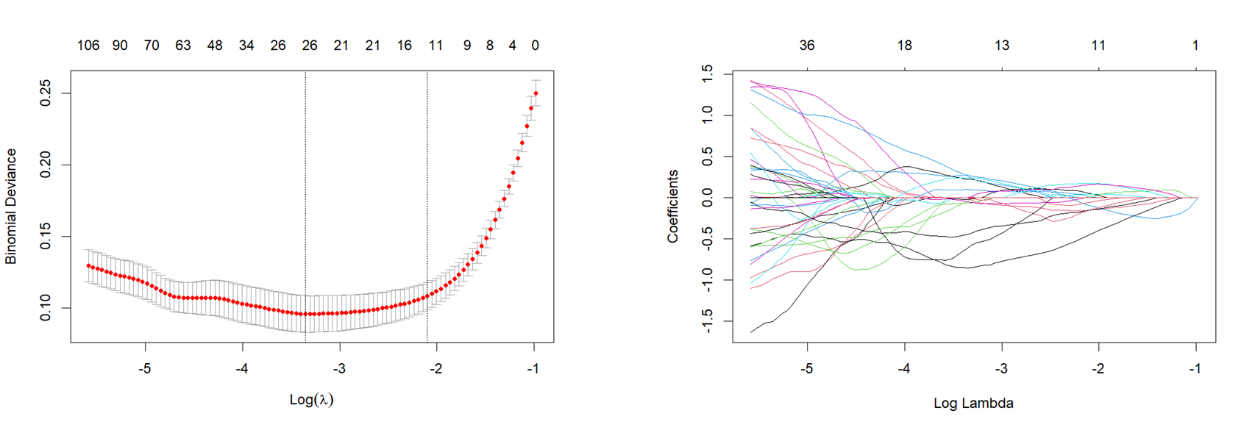


**Supplementary Figure 4.** Calculation of the LASSO logistic regression algorithm in Resnet50 Feature.


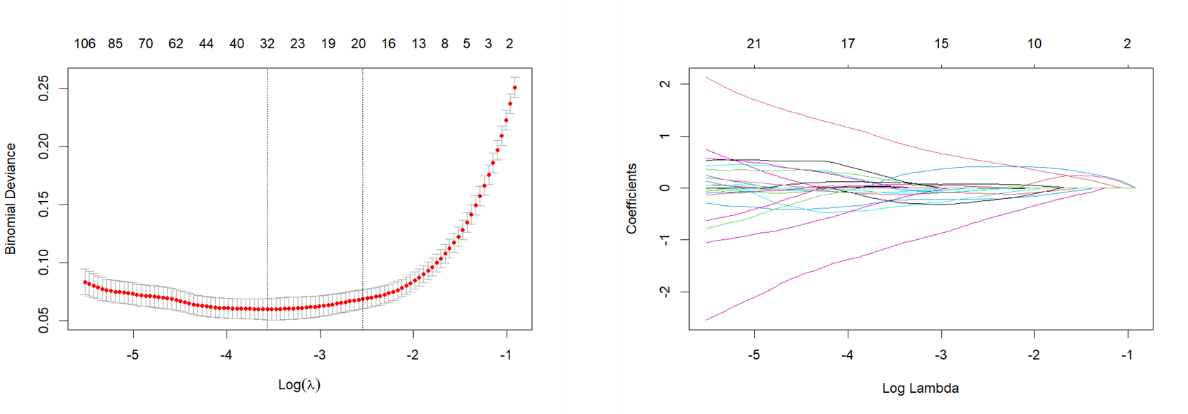


**Supplementary Figure 5.** Calculation of the LASSO logistic regression algorithm in ConvNext Feature.


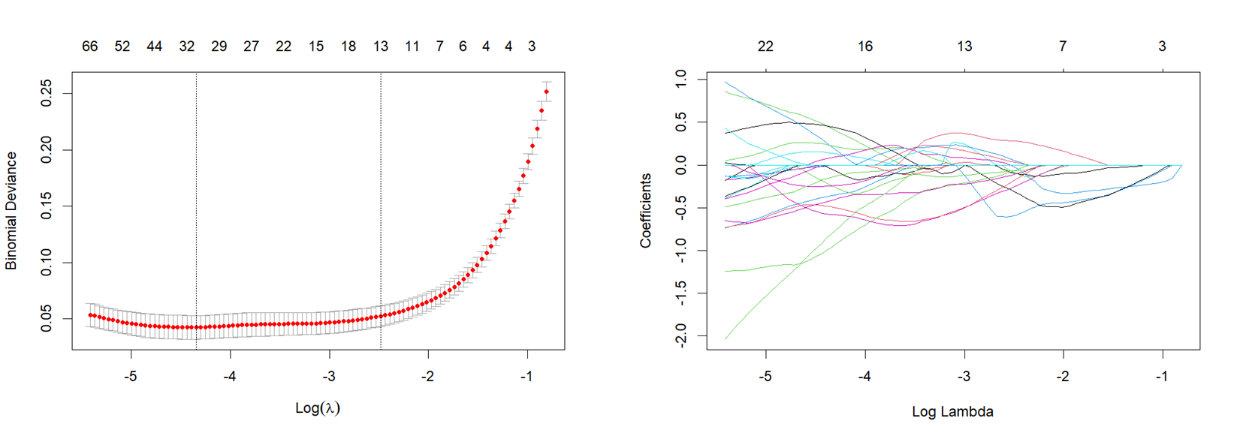


**Supplementary Figure 6.** Calculation of the LASSO logistic regression algorithm in Swin Transformer Feature.


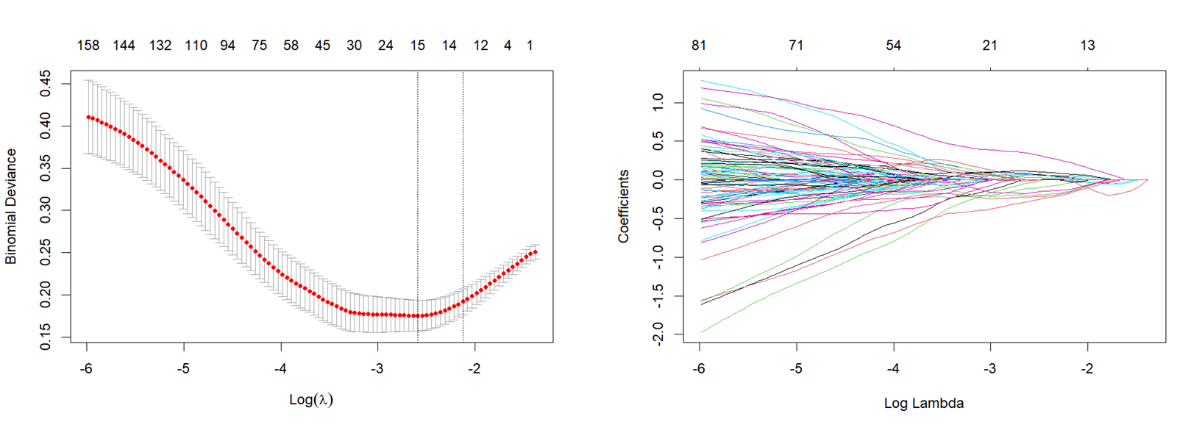


**Supplementary Figure 7.** Calculation of the LASSO logistic regression algorithm in EfficientNet Feature.
