# Supplementary material for: An Integrated Clinical‐Radiomics‐Deep Learning Model Based on 18F‐FDG PET/CT for Predicting EGFR Mutation Status in Lung Adenocarcinoma
Source: Cancer Med. 2025 Nov 20;14(22):e71370. doi: 10.1002/cam4.71370 (PMC12631541; doi:10.1002/cam4.71370)
Supplement: Supplementary file 3 — Text S1:–S6.cam471370‐sup‐0003‐SupplementaryText.docx. [file CAM4-14-e71370-s002.docx]

**Supplementary Text1.** The final formula of radiomic feature

Radscore = 0.448 + 0.083*PET_Original_GLRLM_LongRunHighGrayLevelEmphasis + 0.011* PET_Original_NGTDM_Complexity + 0.017*PET_Wavelet-LLH_GLRLM_RunVariance + 0.032* Wavelet-LHH_GLSZM_SmallAreaHighGrayLevelEmphasis + 0.02*PET_Wavelet-HLL_GLSZM_ZoneVariance + 0.023*PET_Wavelet-HLH_GLSZM_SmallAreaLowGrayLevelEmphasis + 0.117*PET_Wavelet-HHL_GLSZM_GrayLevelNonUniformity + 0.05*PET_Wavelet-HHH_GLSZM_GrayLevelNonUniformity + 0.127*CT_Original_Shape_Sphericity – 0.021* CT_Original_GLCM_ClusterShade + 0.08*CT_Original_GLRLM_ShortRunHighGrayLevelEmphasis + 0.005*CT_Wavelet-LLH_Firstorder_Skewness + 0.006*CT_Wavelet-HLL_Firstorder_Skewness + 0.019*CT_Wavelet-HLL_GLCM_ClusterShade + 0.071*CT_Wavelet-HHL_Firstorder_Median

**Supplementary Text2.** The final formula of Dense169 feature

Dlscore1 = 0.444 + 0.029*DL19 + -0.02*DL53 + 0.021*DL163 + 0.009*DL168 + -0.018*DL292 + 0.065*DL415 + 0.01*DL418 + 0.003*DL624 + -0.035*DL744 + 0.031*DL808 + 0.014*DL883 + -0.04*DL1125 + 0.044*DL1181 + 0.005*DL1267 + 0.034*DL1332 + 0.026*DL1472

**Supplementary Text3.** The final formula of Resnet50 feature

Dlscore2 = 0.447 + -0.043*DL236 + -0.018*DL258 + 0.015*DL325 + 0.012*DL392 + 0.042*DL504 + -0.002*DL507 + 0.025*DL811 + -0.014*DL817 + 0.015*DL846 + -0.051*DL947 + 0.002*DL949 + -0.006*DL951 + 0.004*DL1009 + 0.018*DL1049 + 0.006*DL1080 + 0.016*DL1203 + -0.005*DL1225 + 0.015*DL1395 + 0.014*DL1447 + 0.014*DL1639 + 0.005*DL1666 + -0.013*DL1668 + 0.019*DL1798 + 0.047*DL1964 + -0.064*DL2001

**Supplementary Text4.** The final formula of ConvNext feature

Dlscore3 = 0.451 + 0.006*DL30 + -0.01*DL91 + 0.009*DL92 + -0.018*DL143 + -0.007*DL256 + -0.026*DL264 + -0.002*DL267 + -0.005*DL272 + 0.001*DL274 + -0.001*DL318 + 0.007*DL334 + 0.012*DL375 + -0.015*DL447 + 0.03*DL538 + -0.056*DL542 + 0.084*DL544 + 0.012*DL572 + -0.018*DL630 + -0.021*DL633 + -0.033*DL651 + 0.024*DL672 + -0.039*DL751 + -0.037*DL804 + 0.031*DL851 + -0.035*DL863 + -0.005*DL941 + -0.004*DL959 + 0.014*DL964 + 0.019*DL971

**Supplementary Text5.** The final formula of Swin Transformer feature

Dlscore4 = 0.447 + 0.001*DL31 + 0.028*DL65 + -0.05*DL92 + -0.04*DL138 + 0.014*DL161 + -0.017*DL214 + 0.001*DL322 + 0.016*DL329 + 0.058*DL359 + 0.02*DL439 + 0.003*DL478 + 0.004*DL480 + 0.024*DL503 + 0.03*DL527 + 0.019*DL548 + -0.002*DL567 + 0.025*DL585 + -0.036*DL609 + -0.016*DL622 + -0.021*DL630 + -0.014*DL659 + -0.012*DL678 + -0.035*DL728 + -0.01*DL740 + -0.028*DL837 + -0.028*DL838 + 0.025*DL856 + -0.005*DL893 + 0*DL958 + -0.031*DL975 + -0.012*DL984 + 0.032*DL986 + -0.03*DL987

**Supplementary Text6.** The final formula of EfficientNet feature

Dlscore15 = 0.441 + 0.015*DL99 + 0.021*DL309 + -0.005*DL317 + 0.016*DL330 + 0.002*DL362 + -0.037*DL413 + 0.009*DL522 + 0.022*DL660 + -0.024*DL663 + -0.055*DL876 + -0.048*DL926 + 0.063*DL1010 + -0.034*DL1266
